# Accuracy of two different imageless navigation systems for leg length and global offset change in total hip arthroplasty: A comparison using two-dimensional radiographic and three-dimensional CT-based evaluation

**DOI:** 10.1007/s00264-026-06849-5

**Published:** 2026-05-15

**Authors:** Shine Tone, Yohei Naito, Gai Kobayashi, Hironori Unno, Akihiro Sudo, Masahiro Hasegawa

**Affiliations:** 1 Present Address: Department of Orthopaedic Surgery, Mie University, Graduate School of Medicine, Tsu, Japan; 2 Yonaha Okanoue Hospital, Kuwana, Japan

**Keywords:** Total hip arthroplasty, Imageless navigation, Large-console, Portable, Leg length change, Global offset change

## Abstract

**Purpose:**

This study aimed to evaluate the accuracy of two imageless navigation systems for restoring leg length change (LLC) and global offset change (GOC) in total hip arthroplasty (THA) using two-dimensional (2D) radiographic and three-dimensional computed tomography (3D CT)-based assessment methods.

**Methods:**

Patients undergoing primary cementless THA were divided into two groups based on the imageless navigation system used: a large-console group (*n* = 120) and a portable handheld group (*n* = 83). Intraoperative navigation measurements of the LLC and GOC were compared with values derived from preoperative and postoperative assessments, and absolute measurement errors were calculated. Accuracy was evaluated using 2D radiographic and 3D CT-based measurements. Between-system differences and discrepancies between 2 and 3D assessment methods were analyzed.

**Results:**

Absolute LLC error in the large-console group was 2.7 ± 3.3 mm on 2D radiographic evaluation and 2.5 ± 3.3 mm on 3D CT-based evaluation, compared with 2.9 ± 2.7 mm and 3.0 ± 2.8 mm, respectively, in the portable handheld group. LLC error was significantly lower in the large-console group on 3D evaluation (p = 0.004). Absolute GOC error did not differ significantly between groups. No differences were observed between 2 and 3D evaluations for LLC, whereas most GOC-related parameters differed significantly between methods.

**Conclusion:**

Imageless navigation systems achieved favorable accuracy for LLC and GOC in THA. While radiographic assessment is sufficient for evaluating leg length, 3D CT-based evaluation provides a more consistent and less position-dependent assessment of global offset.

## Introduction

Total hip arthroplasty (THA) is a well-established surgical intervention for hip disorders that provides reliable pain relief and functional improvement [1]. The primary goal of total hip arthroplasty (THA) is to restore normal hip biomechanics through accurate implant positioning, including precise restoration of leg length and global offset. Computer-assisted surgery (CAS), including navigation and robotic systems, has been introduced to improve surgical precision and has been associated with improved acetabular cup positioning and fewer postoperative complications [2–4].

Leg length discrepancy (LLD) has been associated with patient dissatisfaction, gait disturbance, and litigation [5–7], whereas insufficient restoration of hip offset may adversely affect dislocation risk, abductor muscle strength, impingement, and polyethylene wear [8–12]. Although CAS has been shown to improve cup positioning and leg length restoration, evidence for offset restoration remains limited, partly due to the difficulty of accurate postoperative assessment. Conventional two-dimensional (2D) radiographic measurements are affected by limb positioning and femoral anteversion, particularly in offset evaluation [13, 14]. This study quantitatively evaluated the accuracy of two different imageless navigation systems in restoring leg length and hip offset in THA using both 2D and three-dimensional (3D) assessment methods. We hypothesized that (1) portable handheld imageless navigation would achieve an accuracy comparable to that of large-console imageless navigation in restoring leg length and hip offset, and (2) 3D assessment could more precisely detect navigation-related reconstruction errors than 2D radiographic assessment, particularly for offset restoration.

## Patients and methods

### Patient selection

This retrospective observational cohort study was approved by the institutional review board (No. H2018-083) and conducted in accordance with the Declaration of Helsinki [15]. Patients were divided into two groups based on the navigation system used during each study period: cementless THA performed using a large-console imageless navigation system between June 2014 and April 2019 (large-console group) and cementless THA performed using a portable handheld imageless navigation system between April 2020 and June 2022 (portable handheld group). Cases were excluded because of the use of implants from manufacturers that did not support intraoperative leg length and offset measurements (*n* = 180), unsuccessful or unreliable hip center registration (*n* = 5), incomplete postoperative imaging or essential navigation data (*n* = 24), and THA performed following prior osteotomy (*n* = 5). A total of 220 hips were included in the analysis. Demographic data, including age, sex, body mass index, diagnosis, and surgical approach, were collected.

### Surgical procedure

All implants were supplied by the same manufacturer (Kyocera, Kyoto, Japan) and consisted of contemporary cementless designs. In the large-console group, acetabular components included AHFIX Q3 (*n* = 36) and SQRUM TT (*n* = 84), whereas all patients in the portable group received the SQRUM TT cup. Femoral stems included J-Taper (*n* = 56) and INITIA (*n* = 64) in the large-console group, and INITIA in all portable cases. Zirconia-toughened alumina heads were used in all cases.

All THAs included in this study were performed in the lateral decubitus position using either a superior or a posterior approach [16]. All procedures were performed by two experienced hip surgeons, each with more than 20 years of clinical experience, who had been performing navigation-assisted THA for over 10 years prior to the study period. The same surgical workflow was used throughout the study.

**Fig. 1:**
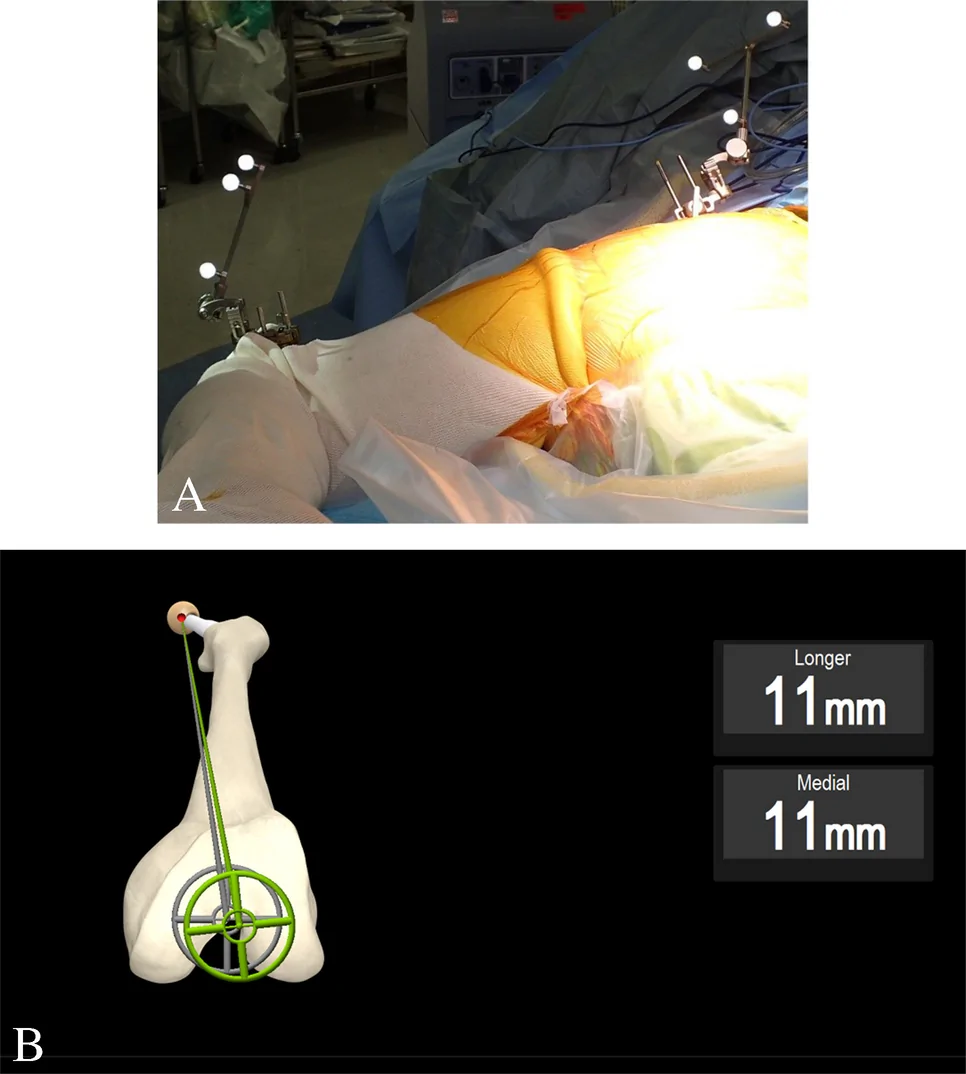
**A-B** Intraoperative workflow of large-console imageless navigation during total hip arthroplasty. (**A**) Pelvic and femoral tags shown in situ after fixation. (**B**) Measurement of leg length change and global offset change was performed using a large-console imageless navigation device

The large-console imageless navigation system used in the large-console group was the KICK Hip application system (BrainLab, Helmstetten, Germany), which has been reported to provide high accuracy in acetabular cup placement and leg length restoration [17, 18]. This system requires the insertion of two reference pins into the pelvis and two pins into the distal femur, to which optical trackers are attached (Fig. 1A). The anterior pelvic plane was used as the pelvic reference plane and was established by palpation of both the anterior superior iliac spines using a tracked pointer. The femoral reference plane was created by registering the piriformis fossa, medial and lateral femoral epicondyles, and ankle center, with the limb held in a neutral extended position. After the final reconstruction, the limb was returned to the preoperatively registered neutral position by aligning the crosshair displayed on the monitor, allowing intraoperative measurements of changes (Fig. 1B). A unique feature of this system is that changes are displayed only when the limb is repositioned within ± 5° of the registered neutral position, which enhances reproducibility of limb positioning and may contribute to improved measurement accuracy. The intraoperative leg length change (LLC) was measured as displacement in the craniocaudal direction, whereas the intraoperative global offset change (GOC) was measured as displacement in the mediolateral direction relative to the preoperative reference planes.

**Fig. 2:**
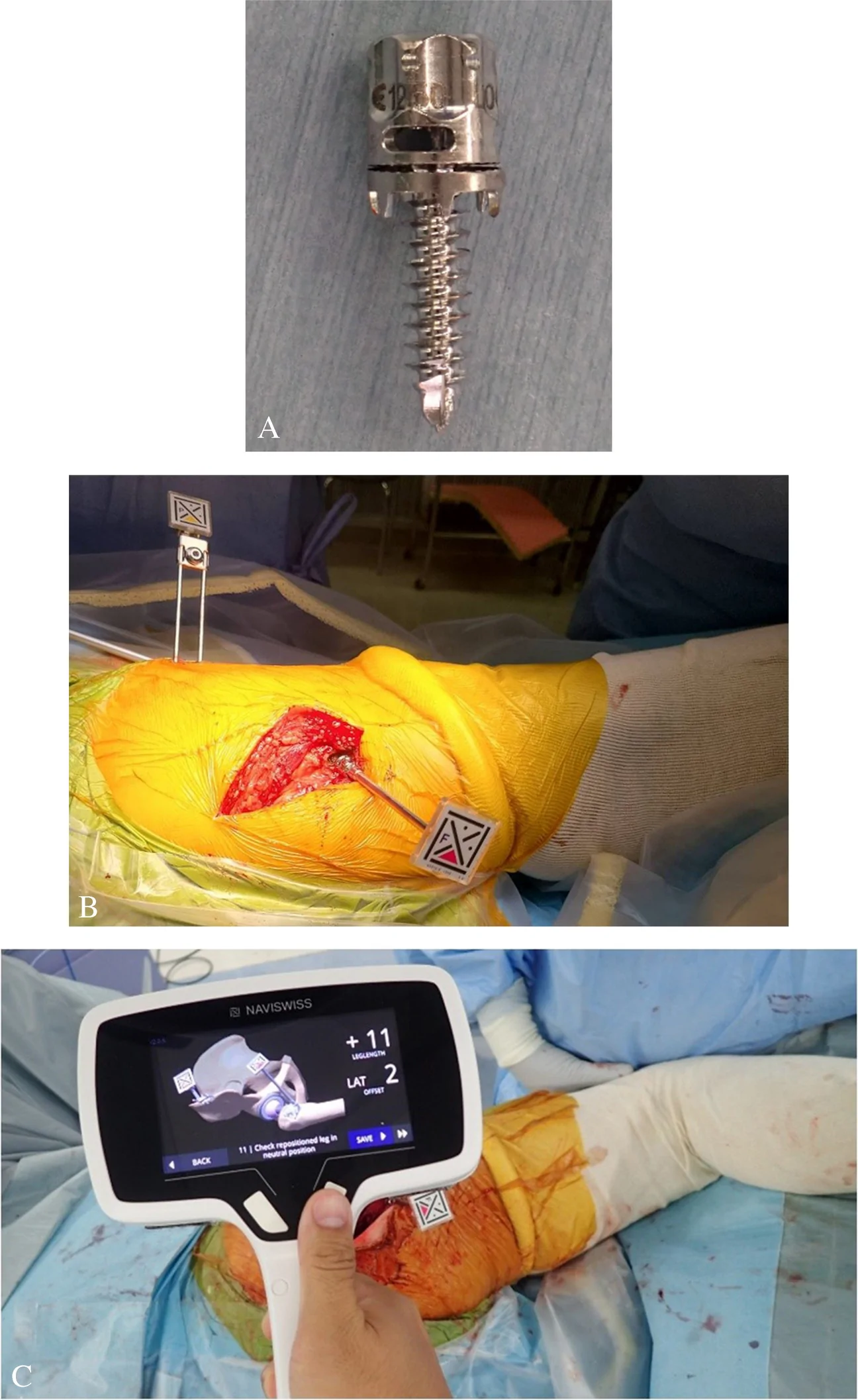
**A-C** Intraoperative workflow of portable handheld imageless navigation during total hip arthroplasty. (**A**) Femoral fixation pin inserted posterior to the greater trochanter for placement of the femoral tag. (**B**) Pelvic and femoral tags shown in situ after fixation. (**C**) Measurement of leg length change and global offset change was performed using a portable handheld imageless navigation device

The portable handheld imageless navigation system used in this study was Naviswiss (Naviswiss AG, Brugg, Switzerland), which has been reported to provide highly accurate acetabular cup positioning [19, 20]. For this system, two 3.0-mm pins were inserted into the iliac crest to secure the pelvic tag, and a femoral fixation pin was inserted posterior to the greater trochanter to attach the femoral tag (Fig. 2A, B). After registering the limb in a neutral extended position, the hip joint was circumducted to register the hip centre. Measurements were obtained after reconstruction, with the limb repositioned to the same neutral position used during registration (Fig. 2C). A key feature of this system is that the femoral reference plane is established using three reference points consisting of the pelvic tag, femoral tag, and registered hip centre, thereby minimizing the influence of limb positioning on measurement accuracy. Changes were defined as the displacement of the femoral tag relative to the preoperative femoral reference plane in the neutral position. The change in the intraoperative LLC was measured in the craniocaudal direction, whereas the intraoperative GOC was measured in the mediolateral direction.

## Radiological analysis

All patients included in the study underwent preoperative and postoperative radiographic and computed tomography (CT) examinations to assess LLC and GOC within two weeks after surgery. All measurements were performed by two independent observers, and interobserver reliability was assessed. Standard anteroposterior pelvic radiographs were obtained in the supine position with both lower limbs internally rotated and the great toes touching the center of the patellae. 2D LLD was measured according to the method described by Ranawat et al. [5] as the distance between a horizontal line connecting both teardrops and the medial apex of the lesser trochanter.

The 2D offset measurements were performed as described by Flecher et al. [21]. The acetabular offset was defined as the perpendicular distance from the center of rotation of the femoral head to the vertical trans-teardrop line, whereas the femoral offset was defined as the distance from the femoral head center to the central axis of the femur. GOC was calculated as the sum of the acetabular and femoral offsets.

Helical CT scans with a slice thickness of 1 mm were obtained from the anterior superior iliac spine to the knee. 3D measurements were performed using a 3D Template system (ZedHip; LEXI Co., Ltd., Tokyo, Japan) after repositioning of the functional pelvic plane [18, 22]. The 3D LLD was defined as the distance between the anterior superior iliac spine and femoral intercondylar fossa. LLC was calculated as the postoperative LLD minus the preoperative LLD for both 2D and 3D assessments. 3D acetabular offset was defined as the perpendicular distance from the femoral head centre to the teardrop line in the functional pelvic plane. The femoral offset was defined as the perpendicular distance from the femoral head center to a line passing through the centres of two circles fitted within the proximal femoral medullary canal. GOC was calculated as the postoperative global offset minus the preoperative global offset for both the 2D and 3D assessments. Increases in LLC and GOC were defined as positive values.

### Statistical analysis

All statistical analyses were performed using EZR (Saitama Medical Center, Jichi Medical University, Saitama, Japan). The normality of the data distribution was assessed using the Kolmogorov–Smirnov test. For normally distributed data, the Student’s t-test was used, and for non-normally distributed data, the Mann–Whitney U test was applied for between-group comparisons. A paired Student’s t-test or Wilcoxon signed-rank test was used for within-group comparisons, as appropriate. Spearman’s rank correlation coefficient was used to assess the correlations. A *p*-value < 0.05 was considered statistically significant.

An a priori power analysis was performed using G*Power version 3.1.9 (University of Kiel, Germany). Based on the effect sizes reported in previous studies [23], a minimum sample size of 76 hips was required to achieve 80% statistical power, with an alpha level of 0.05.

Measurement reproducibility was assessed for both 2D and 3D evaluations using intraclass correlation coefficients (ICCs). For intraobserver reliability, one orthopaedic surgeon (ST) measured each parameter twice in 30 hips at intervals of at least four weeks. For interobserver reliability, two orthopaedic surgeons (ST and YN) independently measured each parameter twice in 30 hips at intervals of at least four weeks. ICC values were interpreted as follows: 0.81–1.00, excellent; 0.61–0.80, substantial; 0.41–0.60, moderate; 0.21–0.40, fair; and 0.00–0.20, poor.

## Results

Intraobserver and interobserver reliability were excellent for all parameters, with higher ICCs for 3D than for 2D measurements.

**Fig. 3:**
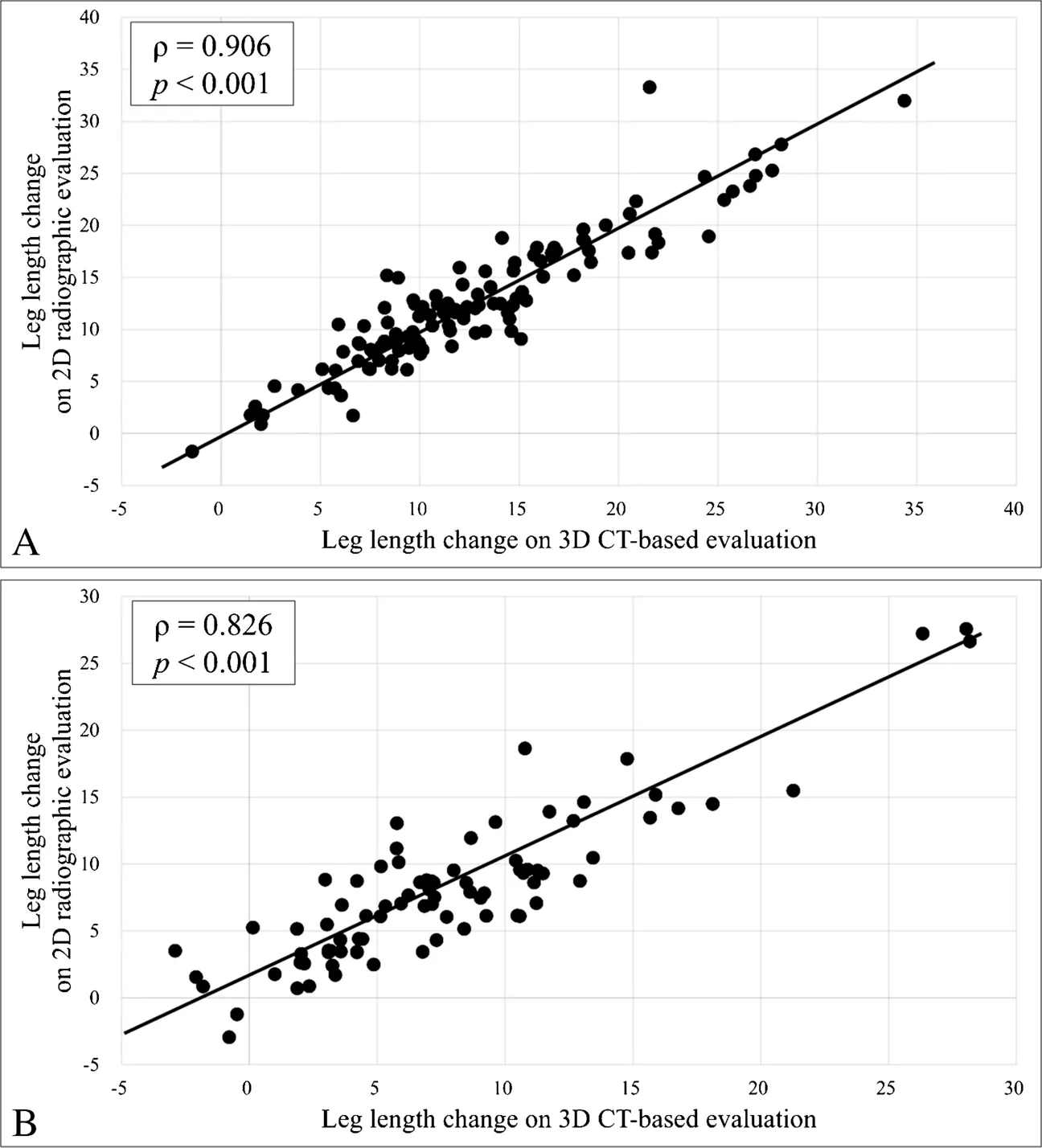
**A-B** Correlation between two-dimensional (2D) radiographic and three-dimensional (3D) computed tomography (CT)-based evaluations of leg length change. (**A**) Large-console imageless navigation; (**B**) Portable handheld imageless navigation

**Fig. 4:**
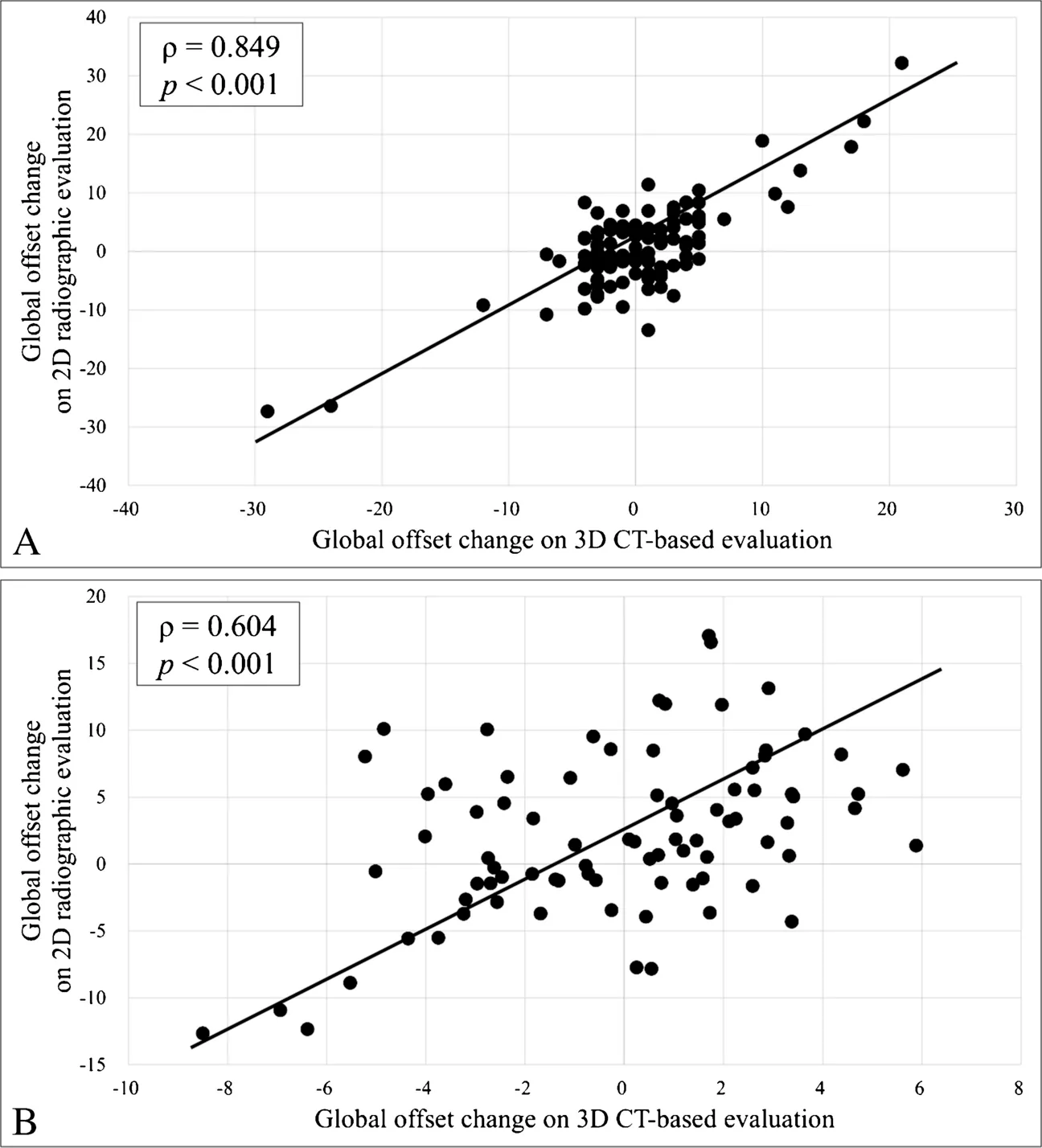
**A-B** Correlation between two-dimensional (2D) radiographic and three-dimensional (3D) computed tomography (CT)-based evaluations of global offset change. (**A**) Large-console imageless navigation; (**B**) Portable handheld imageless navigation

The large-console and portable groups included 120 and 100 hips, respectively. Femoral pin loosening occurred in 17 hips (17%) in the portable group, leaving 83 hips for analysis. Demographics were comparable between groups, except for the surgical approach (Table 1). Significant correlations were observed between the intraoperative navigation measurements and both 2D radiographic and 3D CT-based evaluations of LLC and GOC in both groups (Figs. 3 and 4). However, the correlation for GOC on 2D radiographic evaluation was slightly weaker than that for the other parameters.

**Table 1:** Demographic data of included patients

	Large-console group *n* = 120	Portable handheld group *n* = 83	*p* value
Age at operation, years (mean ± SD)	69.1 ± 9.4	70.7 ± 8.7	0.352
Sex, *n* (male/female)	24/94	16/67	1
Body mass index, kg/m^2^ (mean ± SD)	23.9 ± 4.4	24.6 ± 3.9	0.05
Operated side, *n* (left/right)	67/53	39/44	0.253
Bilateral hip disease, *n* (%)	68 (56.7)	51 (61.5)	0.563
Diagnosis			
Primary osteoarthritis, *n*	19	10	0.795
Secondary osteoarthritis, *n*	89	66	
Crowe classification, *n* (Ⅰ/Ⅱ/Ⅲ/Ⅳ)	75/11/3/0	62/1/3/0	
Osteonecrosis, *n*	9	6	
Rheumatoid arthritis, *n*	3	1	
Surgical approach, *n* (superior/posterior)	19/101	64/19	< 0.001

A comparison of the measurement accuracies of the two navigation systems is presented in Table 2. No significant differences were observed between the groups for any parameter on 2D radiographic evaluation. In the 3D CT-based evaluation, the absolute LLC error was significantly smaller in the large-console group than in the portable handheld group. Although the absolute GOC error did not differ significantly between the groups, the proportion of cases with an absolute GOC error within 10 mm was significantly higher in the portable handheld group.

**Table 2:** Comparison of two-dimensional (2D) radiographic and three-dimensional (3D) computed tomography-based accuracy of leg length change (LLC) and global offset change (GOC) between large-console and portable handheld imageless navigation groups

Measurement	Variable	Large-console group (mean ± SD or %) *n* = 120	Portable handheld group (mean SD or %) *n* = 83	*p*-value
2D measurement	Absolute LLC error (mm)	2.7 ± 3.3	2.9 ± 2.7	0.196
	Absolute LLC error within 3 mm (%)	69.2	63.8	0.236
	Absolute LLC error within 5 mm (%)	90.0	86.0	0.829
	Absolute LLC error within 10 mm (%)	96.7	97.6	1
	Absolute GOC error (mm)	5.0 ± 5.4	5.0 ± 4.0	0.582
	Absolute GOC error within 3 mm (%)	46.7	37.3	0.198
	Absolute GOC error within 5 mm (%)	66.7	56.6	0.184
	Absolute GOC error within 10 mm (%)	90.8	86.7	0.368
3D measurement	Absolute LLC error (mm)	2.5 ± 3.3	3.0 ± 2.8	0.004
	Absolute LLC error within 3 mm (%)	75.8	67.5	0.204
	Absolute LLC error within 5 mm (%)	90.0	89.2	1
	Absolute LLC error within 10 mm (%)	96.7	96.4	1
	Absolute GOC error (mm)	3.6 ± 4.6	2.5 ± 1.7	0.240
	Absolute GOC error within 3 mm (%)	70.0	69.9	0.755
	Absolute GOC error within 5 mm (%)	87.5	90.4	0.492
	Absolute GOC error within 10 mm (%)	92.5	100	0.012

*LLC* Leg length change, *GOC* Global offset change.

Values are presented as mean ± standard deviation or percentage (number of hips). Absolute LLC and GOC errors were defined as the absolute differences between the intraoperative navigation measurements and the corresponding changes calculated from the preoperative and postoperative measurements.

Comparisons between the 2D radiographic and 3D CT-based evaluations in both groups are summarized in Table 3. No significant differences were observed in leg length-related parameters. In contrast, most global offset-related parameters showed significant differences.

**Table 3:** Two-dimensional (2D) radiographic and 3-dimensional (3D) computed tomography-based measurements of leg length- and global offset-related parameters in the large-console and portable handheld groups

	Variable	2D measurement (mean ± SD or %)	3D measurement (mean ± SD or %)	*p*-value
Large-console group	Mean preoperative LLD (mm)	− 10.4 ± 8.5	− 10.5 ± 9.1	0.986
	Mean postoperative LLD (mm)	1.5 ± 5.5	2.3 ± 6.7	0.887
	Mean LLC (mm)	11.9 ± 6.4	12.8 ± 6.4	0.175
	Absolute LLC error (mm)	2.7 ± 3.3	2.5 ± 3.3	0.121
	Absolute LLC error within 3 mm (%)	69.2	75.8	0.312
	Absolute LLC error within 5 mm (%)	90.0	90.0	1
	Absolute LLC error within 10 mm (%)	96.7	96.7	1
	Mean preoperative GOD (mm)	1.7 ± 6.4	4.9 ± 6.2	< 0.001
	Mean postoperative GOD (mm)	− 3.7 ± 8.3	− 0.8 ± 7.6	< 0.001
	Mean GOC (mm)	− 5.4 ± 9.1	− 5.7 ± 8.6	0.215
	Absolute GOC error (mm)	5.0 ± 5.4	3.6 ± 4.6	< 0.001
	Absolute GOC error within 3 mm (%)	46.7	70.0	< 0.001
	Absolute GOC error within 5 mm (%)	66.7	87.5	< 0.001
	Absolute GOC error within 10 mm (%)	90.8	92.5	0.816
Portable handheld group	Mean preoperative LLD (mm)	− 5.9 ± 9.4	− 5.3 ± 13.8	0.897
	Mean postoperative LLD (mm)	2.1 ± 6.9	2.5 ± 12.1	0.506
	Mean LLC (mm)	8.0 ± 5.7	7.7 ± 6.1	0.563
	Absolute LLC error (mm)	2.9 ± 2.7	3.0 ± 2.8	0.843
	Absolute LLC error within 3 mm (%)	63.8	67.5	0.744
	Absolute LLC error within 5 mm (%)	86.0	89.2	1
	Absolute LLC error within 10 mm (%)	97.6	96.4	1
	Mean preoperative GOD (mm)	1.0 ± 5.6	4.0 ± 6.0	< 0.001
	Mean postoperative GOD (mm)	0.5 ± 6.4	1.2 ± 5.6	0.172
	Mean GOC (mm)	− 0.5 ± 7.0	− 2.9 ± 5.9	< 0.001
	Absolute GOC error (mm)	5.0 ± 4.0	2.5 ± 1.7	< 0.001
	Absolute GOC error within 3 mm (%)	37.3	69.9	< 0.001
	Absolute GOC error within 5 mm (%)	56.6	90.4	< 0.001
	Absolute GOC error within 10 mm (%)	86.7	100	< 0.001

*LLD* Leg length discrepancy, *LLC* Leg length change, *GOD* Global offset discrepancy, *GOC* Global offset change.

Values are presented as mean ± standard deviation (SD) or percentage (number of hips). Absolute LLC and GOC errors were defined as the absolute differences between the intraoperative navigation measurements and the corresponding changes calculated from the preoperative and postoperative measurements.

## Discussion

One of the major strengths of the present study is the comprehensive assessment of navigation accuracy for both LLC and GOC using 2D radiographic evaluation and 3D CT-based evaluation, which are widely used and considered more precise. This dual approach enabled not only direct comparison between different navigation systems but also clarification of methodological differences between conventional 2D and advanced 3D assessment techniques. A key finding of this study was that no significant difference was observed between 2 and 3D evaluations regarding LLC accuracy, whereas GOC was more consistently assessed by 3D CT-based evaluations than by conventional radiographic measurements. To the best of our knowledge, no previous study has simultaneously assessed leg length and global offset using 2D and 3D methodologies in navigation-assisted THA. Therefore, the present study provides novel insights into not only navigation performance but also optimal evaluation methods for assessing leg length and global offset restoration. A notable limitation of the present study is the relatively high rate of femoral fixation pin loosening in the portable handheld navigation system, which resulted in exclusion of 17% of cases from the accuracy analysis. This finding highlights not only a potential source of measurement bias but also an important practical limitation of the system in real-world clinical use. Therefore, these results should be interpreted as reflecting the accuracy of the system under successful fixation conditions rather than overall system reliability. Furthermore, the present findings should be interpreted cautiously, as the study design does not allow for a direct comparison of the intrinsic performance of the two navigation systems.

LLC is commonly assessed using radiographs, which are influenced by limb positioning, whereas CT-based 3D assessment is less position-dependent [24–29]. Most previous studies evaluated navigation accuracy by comparing preoperative planning with postoperative outcomes, and relatively few reported absolute navigation errors. Previous studies have reported absolute LLC errors of approximately 1.7–4 mm [30–35], and similar results were observed in the present study. In this study, 3D evaluation showed smaller LLC errors in the large-console system than in the portable system. However, as no differences were observed between 2 and 3D assessments within each system, radiographic evaluation appears sufficient for routine clinical assessment. These findings should be interpreted cautiously given the lack of consistent differences across evaluation methods.

In contrast, substantial discrepancies were observed between the 2D and 3D evaluations in the assessment of GOC. Previous studies have demonstrated that 2D evaluations tend to underestimate offset changes compared with 3D CT-based assessments [22]. Consistent with these findings, the present study revealed marked differences in the absolute GOC error between 2 and 3D evaluations, as well as stronger correlations between intraoperative navigation values and postoperative measurements when assessed using 3D methods for both navigation systems. Prior studies using 2D radiographic assessment have shown wide variability, ranging from approximately 1 to 5 mm, whereas studies employing 3D CT-based evaluation have consistently reported more precise restoration within approximately 2 to 3 mm [30, 34–36]. The 3D-based results observed in the present study closely align with these previous reports, further supporting the reliability of CT-based assessment for global offset evaluation. Future research should clarify the relationship between global offset restoration and clinical outcomes. As most existing studies rely on 2D radiographic measurements, further research using CT-based assessment is warranted.

This study has several limitations. First, CT-based 3D evaluation was used, which involves higher radiation exposure than radiography [37, 38]. However, it allows precise anatomical reconstruction and reduces the influence of limb positioning. Given that radiographic evaluation may be insufficient for accurate assessment of global offset, CT-based analysis was necessary to achieve the study objectives. Second, this was a single-centre, retrospective, nonrandomized study, which may limit generalizability and introduce selection bias. Although surgical approach differed between groups, all procedures were performed in the lateral decubitus position, minimizing its impact on outcomes. Third, in the portable handheld group, 17% of cases were excluded due to femoral pin loosening, which may affect measurement reliability and reflects a limitation of the system. Finally, clinical outcomes were not evaluated. Further studies are needed to determine whether improved offset restoration translates into better clinical outcomes.

## Conclusions

This study demonstrated that both large-console and portable handheld imageless navigation systems achieved favorable accuracy in leg length and global offset restoration. Although LLC can be adequately evaluated using conventional radiographic assessment, 3D CT-based evaluation may provide a more consistent and less position-dependent assessment of GOC. These findings may have implications for the selection of postoperative evaluation methods in navigation-assisted THA.

## Data Availability

No datasets were generated or analysed during the current study.
